# Anesthesiologists’ Perspectives on GLP-1 Receptor Agonists in Elective Surgeries: A Qualitative Survey Analysis of National Data

**DOI:** 10.7759/cureus.95986

**Published:** 2025-11-03

**Authors:** Benjamin Boudreau, Nicholas C Watson

**Affiliations:** 1 Department of Anesthesiology, Michigan State University College of Human Medicine, Grand Rapids, USA; 2 Critical Care, Anesthesia Practice Consultants, Grand Rapids, USA

**Keywords:** ambulatory anesthesiology, glp-1 receptor agonists, patient safety, perioperative medicine, safety in anesthesiology

## Abstract

Glucagon-like peptide-1 receptor agonists (GLP-1 RAs) are a novel class of drugs that have recently gained popularity for their ability to effectively manage type 2 diabetes mellitus and promote weight loss by delaying gastric emptying. The American Society of Anesthesiologists published consensus-based guidance in 2023 for the preoperative management of patients taking GLP-1 RAs. Currently, the literature to support best practice is sparse. Therefore, the purpose of this study is to gather and analyze the perspectives of currently practicing anesthesiologists regarding their experiences providing care for patients taking GLP-1 RAs and their thoughts concerning the American Society of Anesthesiologists (ASA)’s 2023 guidance. This survey study was assigned exempt status by the Michigan State University Institutional Review Board (IRB). A focus group of six practicing anesthesiologists provided free-text statements describing their thoughts and experience with GLP-1 RAs, from which common themes and preferred professional language were identified and used to develop survey items. An iterative expert validation and cognitive pretesting process was used, whereby the focus group provided feedback on draft survey items, items were edited, and the focus group re-reviewed the items repeatedly until all survey items were accurately reflective of the target research questions set by the authors. The final survey was hosted in Qualtrics software, and the survey invitation link was sent to the electronic mailing list for the author's (NCW) practice and to leadership contacts at 27 private and academic practices. The survey was available in June 2024 and remained open for responses for 11 weeks. A total of 236 responses completed the survey, with 61 responses from the author’s (NCW) practice (response rate of 51.6%) and 175 responses from the national distribution. Most anesthesiologists were in their late or early careers, and 65% see patients on a GLP-1 RA at least once per week. Moreover, 66% of physicians have read the ASA’s 2023 guidance document, and 66% of physicians agreed or strongly agreed that the ASA’s 2023 guidance is effective at preserving patient safety. In a free-response question, most respondents commented on the 2023 guidance’s lack of conclusive evidence, uncertainty of when to hold weekly GLP-1 doses, and ambiguity of using the gastric point-of-care ultrasound. This survey indicates that most survey respondents perceive the ASA’s 2023 guidance on GLP-1 RAs during elective surgeries as effective in preserving patient safety. This population of physicians also frequently sees patients on a GLP-1 RA and often experiences these patients being properly instructed on and correctly holding these medications. Free-text responses expressed confusion and highlighted the lack of clinical evidence in the ASA’s 2023 guidance. This survey adds support to the ASA’s 2023 guidance of GLP-1 RAs during elective cases; however, many anesthesiologists expressed safety considerations for these patients, necessitating the need for future research.

## Introduction

Glucagon-like peptide-1 receptor agonists (GLP-1 RAs) are an emerging class of medications that were initially granted approval for the glycemic and metabolic management of patients with type 2 diabetes mellitus. This class of medications utilizes a peptide derived from exendin-4 to promote the activation of the GLP-1 receptor, resulting in widespread activation of the incretin system. Exenatide was the first GLP-1 RA to be approved in the United States in 2005 and was quickly followed by lixisenatide, liraglutide, dulaglutide, and semaglutide in the subsequent decade [1]. In 2021, GLP-1 RAs were approved by the FDA for weight loss, and as of 2024, 12% of Americans self-reported taking a GLP-1 RA [2,3].

Along with their beneficial effects on glycemic control and metabolic disease, GLP-1 RAs have been shown to cause significant gastrointestinal (GI) side effects, with the most common symptoms being abdominal pain, constipation, diarrhea, and nausea/vomiting [4]. In the perioperative setting, GLP-1 RAs have challenged the traditional preoperative fasting protocols as the intended effects of the medications potentially lead to an increased risk of regurgitation and pulmonary aspiration of gastric contents [5].

 In 2023, the American Society of Anesthesiologists (ASA) released “consensus-based guidance” advising anesthesiologists on the optimal preoperative management of patients taking GLP-1 RAs [6]. The ASA advised physicians to follow the current ASA fasting protocols and to hold weekly injections the week before the procedure and daily dosing the day of the procedure. Additional guidance was provided for managing patients presenting with GI symptoms and for patients who did not hold their GLP-1 RA as advised.

The ASA’s 2023 guidance document initiated academic discussion, particularly in the context of limited evidence to support the guidance. As of 2025, there have been no high-quality studies linking GLP-1 RAs to increased intraoperative aspiration risk; however, numerous case reports have been published detailing intraoperative aspiration despite correctly following the 2023 ASA guidance [7,8]. In addition, new evidence from the American Journal of Gastroenterology found no evidence that GLP-1 RAs delay gastric emptying [9]. Until definitive evidence is available to refine best practices, collective clinical experience provides valuable insights into this area of uncertainty. Therefore, the purpose of this study was to identify the experiences and opinions of practicing anesthesiologists regarding GLP-1 RAs in elective procedures.

An abstract for this study was accepted as a poster presentation for the 78th PostGraduate Assembly in Anesthesiology in December 6-9, 2024, in New York, NY.

## Materials and methods

This study was a cross-sectional survey of anesthesiologists and was assigned exempt status from the Michigan State University Institutional Review Board. The consensus-based checklist for the reporting of surveys was used (Appendix A) [10].

Survey development

**Figure 1:**
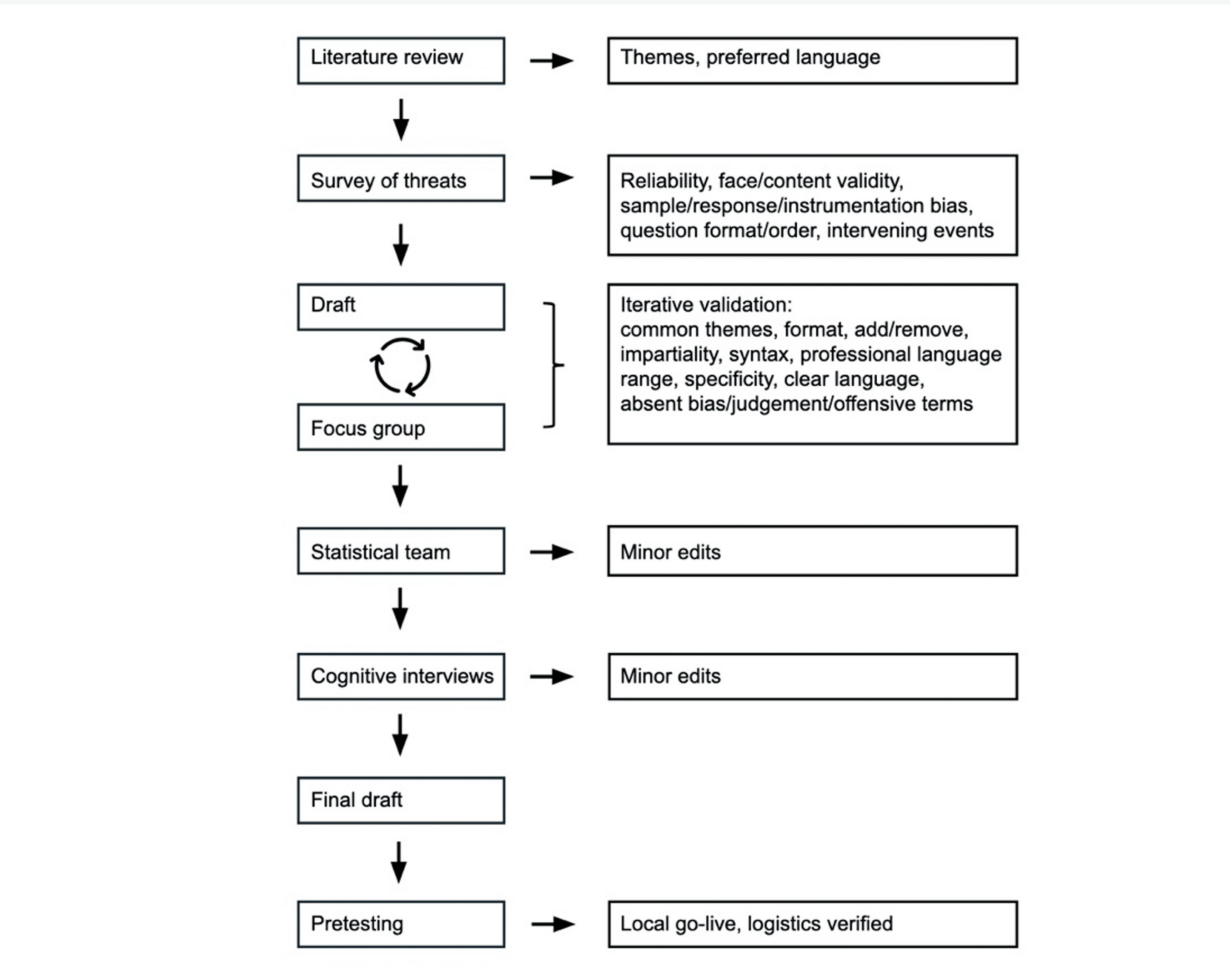
Survey development flowchart

The research survey was developed using best practices, including an expert focus group, iterative expert validation, cognitive interviews, pretesting, and validity and reliability analysis (Figure 1) [11-14]. In addition, we took proactive measures to avoid pitfalls in item development and visual design according to previously published guidance [15,16].

A focus group of six practicing anesthesiologists of varying experience (two to 30 years in practice), gender identity (cis-gender male and cis-gender female), age (30 to 65+ years), and subspecialty practice (general, pediatric, obstetric, critical care) was selected from the author’s (NCW) practice. Focus group members provided free-text statements in response to the request, "Briefly describe the relevance of GLP-1 RAs to anesthesiologists in general and your individual practice." Focus group members were asked to use the same language they would use when discussing these medications with colleagues. From these statements, common themes and preferred professional language were identified. The authors also performed a literature review to broadly identify recurrent themes and terminology regarding GLP-1 RAs (Appendix B).

Next, a large set of potential survey items was drafted using terminology and themes identified in the literature review and in the focus group statements. An iterative expert validation process was used with the aim of assuring that the content and scope of the items adequately addressed the goals of the study. The focus group provided feedback on draft survey items, the items were edited by the authors, and the focus group re-reviewed the items repeatedly until all survey items were relevant and accurate. Focus group members were specifically asked to provide feedback on the following elements: format, items to add, items to remove, the impartiality of each item, whether the language was easily understood and common among anesthesiologists, clarity of syntax, if answer options covered a sufficient range, whether answer options allowed for a clear and specific response, and whether the language of the items was free of commercial bias/judgment/offensive terminology. Great attention was paid to remove any ambiguous or unclear language in the items. The draft survey was then reviewed by the Michigan State University statistical team, and a few minor edits were made based on this feedback.

Cognitive interviews were performed wherein focus group members and four other anesthesiologists not in the focus group were individually asked to state out loud in their own words what each draft survey item stated/asked while an author listened. The purpose of this was to ensure that the items were being interpreted as intended. A few minor edits were made based on this process.

Pretesting was performed by releasing the final version of the survey to a subset of the intended target population prior to widespread release. A convenience sample of 118 anesthesiologists in the author’s (NCW) practice received an email request to complete the online survey. No issues were encountered with the logistics of sending and completing the survey. Initial responses demonstrated that the survey link and automated data collection system functioned appropriately. Anecdotally reported time to complete the survey was less than five minutes.

Multiple strategies were employed to minimize observed and possible threats to the reliability and validity of the survey. After compiling an inventory of threats, solutions were sought for each [11]. Reliability was boosted by using preferred professional language and assuring that all items were interpreted the same way by all respondents during cognitive interviews. Formal reliability testing was not performed because survey items were not summed to an index score, and none of the items were intended to function together. The face validity of items was preserved by assuring that questions pertained to GLP-1 RAs in the context of clinical practice. Face validity also benefited from iterative expert validation to eliminate poorly worded questions. Content validity was achieved by using a literature review and an expert focus group to ensure that the full scope of clinical concerns about GLP-1 RAs was reflected in the survey items.

Administering the survey to anesthesiologists was designed to have the sample population reflect the target audience of the study findings, thus limiting sample bias; however, using a snowball sample may have introduced unknown sampling bias because of the lack of control over who eventually received an invitation and who responded to the survey. Similarly, sampling bias may have been introduced in the pretesting convenience sample, as many of these respondents worked in locations with the same institutional preoperative medication instructions and shared workplace norms. Non-response bias was not measured or controlled because very little information about respondents was collected with the survey (years in practice was the only identity information collected).

The threat of poor question format and the risk of response bias from question ordering effect were mitigated by checking items against published lists of common pitfalls [15,16]. A threat of intervening events affecting survey responses was ameliorated by having a short time between pretesting release and widespread release of survey invitations, using the survey to query the study population only once, and having the survey available for a limited time after solicitation of participants. The risk of instrumentation bias was eliminated by releasing only the final version of the survey and making no subsequent changes during the time the survey was available to participants.

Survey content

The survey consisted of twelve items regarding years of practice, experience with GLP-1 RAs, knowledge of the ASA’s 2023 guidance, degree of agreement with the ASA’s 2023 guidance, barriers to guideline adherence, symptomatology that would lead to case cancellation, and the utility of gastric point-of-care ultrasound (POCUS). An additional item allowing free text response asked participants to comment on any thoughts or concerns they had regarding GLP-1 RAs in elective cases.

Survey administration

The survey was hosted on the Michigan State University server using Qualtrics (monthly product release version June 6, 2024, Qualtrics, Provo, UT [17,18]). The survey invitation link was sent to the electronic mailing list for the author's (NCW) practice and to leadership contacts at 27 private and academic practices in the United States. A snowball sampling technique was used, whereby each survey invitation recipient was requested to forward the survey invitation to anesthesiologist colleagues elsewhere. The survey was available in June 2024 and remained open for responses for 11 weeks. Responses were entirely anonymous, and respondents were allowed one response per link through the Qualtrics software. Unique respondents were determined by allowing only one completed survey per Internet protocol address. There was no safeguard against a respondent submitting more than one survey by accessing from multiple Internet protocol addresses. The investigators were unable to determine any information about the respondents that was not included in the survey items, and the Internet protocol addresses of the respondents were not recorded.

Statistical analysis

The survey response data were analyzed using Qualtrics (monthly product release version June 6, 2024, Qualtrics, Provo, UT) [17,18]. Univariate analysis of responses to each item was reported as count and percentage. None of the items were combined, and no sum scores were calculated. No indices or scales were used. Non-responses and non-response errors were not considered in the analysis. Multivariate analysis was used to assess the correlation of variables of interest (years of experience and frequency of seeing patients on GLP1-RAs) with other survey variables. For each pair of variables, correlation was calculated, and Fisher’s exact test was used to assess the independence between the two variables. Significance was set at p < 0.05.

## Results

A total of 277 respondents completed one or more survey items. Of these, 255 individuals responded to the first question, and 233 individuals completed the survey in its entirety. Sixty-one respondents were from the author’s (NCW) institution, and 216 were from the national sample. The response rate for the pretesting survey was 51.6%. The response rate for the national sample was not calculated because the denominator of survey invitation recipients was unknown. There was no tracking mechanism to tally the number of recipients of forwarded survey invitations. 

Survey respondents were distributed across career stages: early career (40%, <10 years in practice, n = 100), middle career (29%, 11-20 years, n = 75), and late career (31%, >21 years, n = 78). Most anesthesiologists reported seeing patients on a GLP-1 RA at least once per week (65%, n = 164), followed by at least once per month (23%, n = 57), once every few months (9%, n = 23), never (2%, n = 5), and at least once a year (1%, n = 3). Most respondents reported their knowledge of how GLP-1 RAs affect surgical patients as “Intermediate” (47%, n = 119), followed by “somewhat strong” (28%, n = 71), “strong” (14%, n = 35), “somewhat weak” (8%, n = 21), and “weak” (2%, n = 6) (Table 1).

**Table 1:** Demographic data and GLP-1 RA exposure The number of responses per answer is recorded alongside their percent of total responses in parentheses. Questions with multiple-choice answers are labeled as such in the question. The total number of individual responses per question is listed as n_total_.

Q1 - How long have you been practicing as an attending Anesthesiologist? (Please round up)	n_count_ (Percentage)
0-5 years	55 (22%)
6-10 years	45 (18%)
11-15 years	41 (16%)
16-20 years	34 (13%)
21+ years	78 (31%)
	n_total_ = 253
Q2 - How often are you seeing patients who are currently taking a glucagon-like peptide-1 (GLP-1) receptor agonist (RA) (oral or intramuscular)?	
Never	5 (2%)
At least once a year	3 (1%)
At least once every few months	23 (9%)
At least one per month	57 (23%)
At least one per week	164 (65%)
	n_total_ = 252
Q3 - Please rate your current knowledge regarding GLP-1 RAs and their effect on surgical patients.	
Weak	6 (2%)
Somewhat weak	21 (8%)
Intermediate	119 (47%)
Somewhat strong	71 (28%)
Strong	35 (14%)
	n_total_ = 252

Regarding the 2023 ASA’s guidance, most anesthesiologists self-reported that they read the guidance document (66%, n = 165), followed by awareness of the guidance document without explicitly reading it (30%, n = 76). Only a few respondents (4%, n = 10) self-reported being unaware of the guidance document. When asked about which guidance was being followed in their practice (select all that apply), most anesthesiologists reported following the ASA’s 2023 guidance (72%, n = 181), then “local hospital/institutional guidance” (52%, n = 132), then not following any guidance (9%, n = 22). For the item asking if the ASA’s 2023 guidance “preserves patient safety”, most anesthesiologists “agree” (52%, n = 125), followed by “neutral” (21%, n = 50), “strongly agree” (14%, n = 34), “disagree” (10%, n = 24), and “strongly disagree” (3%, n = 7) (Table 2).

**Table 2:** Knoweldge and agreement of the American Society of Anesthesiologists (ASA)'s 2023 guidance The number of responses per answer is recorded alongside their percent of total responses in parentheses. Questions with multiple-choice answers are labeled as such in the question. The total number of individual responses per question is listed as n_total_.

Q4 - Are you up to date on the 2023 American Society of Anesthesiologists' guidance regarding GLP-1 receptor agonists on elective surgical patients?	n_count_ (percentage)
I am unaware of the guidance document.	10 (4%)
I am aware of the guidance document, but have not specifically read it.	76 (30%)
I have read the guidance document.	165 (66%)
	n_total_ = 251
Q5 - For patients who are currently taking a GLP-1 receptor agonist, which guidance do you follow in your practice? (Select all that apply)	
2023 ASA guidance	181 (72%)
Local hospital/institution guidance	132 (52%)
I do not follow any specific guidance.	22 (9%)
	n_total _= 252
Q6 - Please review the above ASA's guidelines for GLP-1 receptor agonists. Do you agree with these guidelines?	
Strongly disagree	7 (3%)
Disagree	24 (10%)
Neutral	50 (21%)
Agree	125 (52%)
Strongly agree	34 (14%)
	n_total_ = 240

When asked about how often patients were properly instructed on holding their GLP-1 RA medication over the past six months, most respondents reported “often” (58%, n = 139), followed by “sometimes” (23%, n = 56), “always” (10%, n = 24), “rarely (4%, n = 10), and then “never” (2%, n = 4). Similarly, when asked if patients are correctly following the holding instructions for GLP-1 RAs, most respondents reported “often” (58%, n = 139), followed by “sometimes” (29%, n = 70), “always” (5%, n = 13), “rarely” (4%, n = 10), and “never” (1%, n = 2) (Table 3).

**Table 3:** Experience with patient instruction for holding GLP-1 RA medication The number of responses per answer is recorded alongside their percent of total responses in parentheses. Questions with multiple-choice answers are labeled as such in the question. The total number of individual responses per question is listed as n_total_.

Q7 - In the past six months, how often have your patients been properly instructed (per ASA guidance) regarding holding glucagon-like peptide-1 receptor agonists (GLP-1 RAs) prior to surgery?	n_count_ (percentage)
Never	4 (2%)
Rarely	10 (4%)
Sometimes	56 (23%)
Often	139 (58%)
Always	24 (10%)
N/A - I have not seen a patient currently taking a GLP-1 RA in the past six months.	7 (3%)
	n_total_ = 240
Q8 - In the past six months, how often have your patients correctly followed the preoperative holding instructions (per ASA guidance) for GLP-1 RAs?	
Never	2 (1%)
Rarely	10 (4%)
Sometimes	70 (29%)
Often	139 (58%)
Always	13 (5%)
N/A - I have not seen a patient currently taking a GLP-1 RA in the past six months.	7 (3%)
	n_total_ = 241

In regard to the barriers anesthesiologists face for properly following the ASA’s 2023 guidance (select all that apply), most responded with “differing interpretations of hospital preoperative instructions” (36%, n = 83), followed by “avoiding case cancellation due to productivity pressure” (34%, n = 79), “avoiding case cancellation due to concern of patient dissatisfaction” (33%, n = 76), “differing interpretations of the ASA guidance” (30%, n = 70), “I have not experienced any barriers” (28%, n = 66), “other” (12%, n = 28), and “avoiding case cancellation to maintain personal income” (4%, n = 9) (Tables 4, 5).

**Table 4:** Self-reported barriers to following the ASA's 2023 guidance The number of responses per answer is recorded alongside their percent of total responses in parentheses. Questions with multiple-choice answers are labeled as such in the question. The total number of individual responses per question is listed as n_total_.

Q9 - What, if any, barriers have you experienced in following the ASA's 2023 guidance on glucagon-like peptide-1 receptor agonists (GLP-1 RAs)? (Please select all that apply)	n_count_ (percentage)
Differing interpretations of the ASA guidance	70 (30%)
Differing interpretations of hospital preoperative instructions	83 (36%)
Avoiding case cancellation to maintain personal income	9 (4%)
Avoiding case cancellation due to concerns of patient dissatisfaction	76 (33%)
Avoiding case cancellation due to productivity pressure	79 (34%)
Other	28 (12%)
I have not experienced any barriers.	66 (28%)
	n_total_ = 232

**Table 5:** "Other" free-text response for Question 9 n_total_ represents the total number of respondents who completed a free-text response for Question 9.

Question 9 "Other"
Case booked within the two-week timeline; therefore, unable to stop medication according to guidelines without case delay
Most of our anesthesiologists are unfamiliar with gastric ultrasound and not interested in learning, sadly.
Surgeon resistance
Lack of evidence other than a guidance document
I have a good reason why I do RSI, unless 10 days or two weeks since the last (weekly) dose.
Overinterpretation of guidelines leading to cancellation of patients without symptoms who have not held meds appropriately - people are cancelling instead of intubating with RSI.
Endoscopists and endocrinologists disagree with the need to hold.
Confusion reigns.
Surgeon insisting on doing the case
Difficulty communicating expectations with patients. Difficulty seeing patients in the pre-op clinic early enough to stop one week ahead. Avoiding case cancellation due to productivity - coming from surgeons, not my concern about my productivity. Poorly worded choice. Guidance from the ASA not strongly enough worded. Not enough but in from surgeons. Patients lie about stopping meds, get meds from non-PCPs.
It's inadequate - I have seen plenty of EGDs at my hospital where there is visual evidence of undigested food piled up in the stomach.
Avoiding cancellation d/t an upset surgeon.
Surgeon booking a case less than a week ahead of time, or a preoperative phone appointment less than a week prior to the surgical date, so inadequate lead time to instruct the patient to properly abstain
The guidelines seem like impractical, academic nonsense.
People’s unfamiliarity with the guidelines
Patients refuse to stop them
Challenges getting the instructions to the patients in a timely manner
Guidance to stop "the week before" can result in [the] patient missing two weeks [depending on] when the surgery is relative to the dose.
Gastric ultrasound is not readily available. Additionally, there isn’t much guidance to have a “hard and fast” cancel for GLP-1 if they have taken it within seven days. It seems case by case. Also, most of what I do is cancer surgery, so delaying it is definitely a risk-benefit discussion with the patient and surgeon (over just RSI).
Avoiding case cancellation due to the urgency of cases
NORA/sedation cases not reviewed by the preoperative testing clinic
Dissatisfaction from prescribing physicians (medical weight loss)
Guidelines are currently based on limited evidence.
Pressure from endoscopy leadership that holding GLP-1s is not necessary.
Weighing the risk of delaying surgery vs. the risk of aspiration
Urgent outpatient cases, such as hand trauma often scheduled with less than a week's notice
LMA vs. ETT decision making, not understanding why we only have to hold one day for those who take it daily and knowing when to cancel
n_total _= 27

The participants were then asked which symptoms would lead them to cancel an elective case if a patient presented without correctly holding their GLP-1 RA intramuscular medication (select all that apply). The most common symptoms leading to case cancellation were vomiting (88%, n = 206), nausea (69%, n = 161), fullness (55%, n = 130), bloating (54%, n = 128), and then upset stomach (45%, n = 106). The lesser selected symptoms were lethargy (16%, n = 38), dizziness (14%, n = 33), other (9%, n = 22), none (6%, n = 15), loss of appetite (5%, n = 12), and headache (1%, n = 3). This same question was asked again, but instead with an oral GLP-1 RA medication (select all that apply). The responses to this question were almost identical to the previous question with the selected symptoms being vomiting (86%, n = 200), nausea (70%, n = 162), fullness (54%, n = 126), bloating (53%, n = 124), then upset stomach (46%, n = 108), lethargy (18%, n = 43), dizziness (14%, n = 32), other (9%, n = 20), loss of appetite (9%, n = 20), none (7%, n = 16), and headache (2%, n = 4) (Tables 6-8).

**Table 6:** Hypothetical clinical scenario where a glucagon-like peptide-1 receptor agonist (GLP-1 RA) was not held The number of responses per answer is recorded alongside their percent of total responses in parentheses. Questions with multiple-choice answers are labeled as such in the question. The total number of individual responses per question is listed as n_total_.

Q10 - For patients who have taken an intramuscular glucagon-like peptide-1 receptor agonist (GLP-1 RA) injection less than one week before surgery, which single or combination of symptoms on the day of surgery would lead you to cancel an elective case? (Select all that apply)	n_count_ (percentage)
Nausea	161 (69%)
Vomiting	206 (88%)
Diarrhea	24 (10%)
Headache	3 (1%)
Loss of appetite	12 (5%)
Upset stomach	106 (45%)
Dizziness	33 (14%)
Lethargy	38 (16%)
Fullness	130 (55%)
Bloating	128 (54%)
Other	22 (9%)
None	15 (6%)
	n_total_ = 235
Q11 - For patients who have taken an oral GLP-1 RA less than one day before surgery, which single or combination of symptoms on the day of surgery would lead you to cancel an elective case? (Select all that apply)	
Nausea	162 (70%)
Vomiting	200 (86%)
Diarrhea	27 (12%)
Headache	4 (2%)
Loss of appetite	20 (9%)
Upset stomach	108 (46%)
Dizziness	32 (14%)
Lethargy	43 (18%)
Fullness	126 (54%)
Bloating	124 (53%)
Other	20 (9%)
None	16 (7%)
	n_total_ = 233

**Table 7:** "Other" free-text responses for Question 10 n_total_ represents the total number of respondents who completed a free-text response for Question 10.

Question 10 “Other”
No experience
Cancel regardless of symptoms, if elective case
Evidence of solids (early or late) on gastric ultrasound
Active vomiting
An airway that might be difficult even with a video scope
Not experienced
Recent addition of a drug or a change of dose associated with the above symptoms
Excessive burping/stomach pain
Correlate with POCUS, not just use one symptom
Positive POCUS
Non-empty stomach on POCUS
Would cancel any elective case if < 1 week, NO symptoms needed.
Gastric ultrasound
Very poorly controlled diabetes (A1C greater than 9) and obesity. Other factors that would already slow gastric emptying, with the GLP1 making it worse.
Depends on other patient factors too
Patient preference
Depending on the case. I have cancelled with no symptoms simply because they took it less than a week prior to the DOS
Gastric ultrasound showing fullness
Especially if these are new or exacerbated symptoms from the baseline of how they feel on these meds
Active reflux/can't lie flat
A combination of any of the above symptoms would then include a discussion with the surgeon about the risks/benefits of delaying the particular surgical case.
Any change from baseline re: constitution of GI symptoms would cause me to question whether elective anesthesia is reasonable.
n_total_ = 22

**Table 8:** "Other" free-text responses for Question 11 n_total_ represents the total number of respondents who completed a free-text response for Question 11.

Question 11 “Other”
No experience
Cancel elective surgery regardless of symptoms
Evidence of solids (early or late) on gastric ultrasound
Active vomiting
Concerning airway
Can't answer
As the above comment
Correlate with POCUS, not just use one symptom
Positive POCUS
Non-empty stomach on POCUS
Wouldn’t do the case if they haven’t followed the instructions
Hypoglycemia
Gastric ultrasound
Same as above
Would cancel elective case even if asymptomatic
Depends on the case
Gastric ultrasound showing fullness
Especially if these are new or exacerbated symptoms from the baseline of how they feel on these meds
Active reflux/can't lie flat
Same as above
n_total_ = 20

When asked about the utility of gastric POCUS to increase the quality of care of patients taking a GLP-1 RA, most respondents reported that they were “somewhat interested” (46%, n = 108), followed by “very interested” (24%, n = 56), “not interested” (20%, n = 47), and “I am currently enrolled in or recently finished a POCUS program” (11%, n = 25) (Table 9). Question 13 was a free-response question where anesthesiologists were invited to add any additional comments they had regarding GLP-1 RAs and the ASA’s 2023 guidance. Responses were mixed with numerous answers discussing the need for more evidence to support the current ASA guidance, the feasibility, inconvenience, and subjectivity of using gastric POCUS, and the lack of evidence to support a one-week or daily hold of GLP-1 RA medications (Table 10).

**Table 9:** Interest in gastric point-of-care ultrasound The number of responses per answer is recorded alongside their percentage of the total responses in parentheses. Questions with multiple-choice answers are labeled as such in the question. The total number of individual responses per question is listed as n_total_.

Q12 - Please identify your interest in gastric point-of-care ultrasound to increase the quality of care in patients taking a Glucagon-like peptide-1 receptor agonist (GLP-1 RA).	n_count_ (percentage)
Not interested	47 (20%)
Somewhat interested	108 (46%)
Very interested	56 (24%)
I am currently enrolled in or have recently finished a POCUS program.	25 (11%)
	n_total_ = 236

**Table 10:** Free-text response for opinions regarding the American Society of Anesthesiologists (ASA)’s 2023 guidance n_total_ represents the total number of respondents who completed a free-text response for Question 13.

Q13 - What additional thoughts or concerns would you like to include regarding the current guidelines for glucagon-like peptide-1 receptor agonists (GLP-1 RAs) in elective cases?
One week from the last weekly dose is not long enough to hold GLP-1 RAs. Many patients' gastric motility does not return for several weeks.
ALL patients who have not held GLP-1 RAs for two weeks for weight loss and one week for diabetes should get a gastric ultrasound. ALL anesthesia providers should become proficient in gastric ultrasound.
More evidence/literature regarding the time period during which patients on GLP-1 RAs are at risk for aspiration due to delayed gastric emptying
Updates, if any, since 2023
I do a gastric POCUS on anyone who did not hold their GLP-1 as directed (or as they should have been directed).
We need more literature beyond expert opinion to develop proper guidelines.
I take a weekly injection. I had a recent surgery at a major center in NY City. I also know for a fact that there have been aspiration cases in NY City (I do not know if at the hospital where I had surgery). But, of note, the hospital where I had surgery instructed me to hold two weekly doses. That suggests they know some aspirations have occurred even when the injection is held for one week. Are the ASA guidelines only one week because the ASA does not want to upset surgeons? I work at a center where instructions are to hold one weekly injection, but the pre-op clinic is great, and 99% of patients are aware of what to do. I wonder if the ASA is hesitant to write a policy that might lead to more cancellations? Nice that you are doing this survey. What we truly need is a study of patients who were intubated after various time frames since the last GLP-1 receptor agonist, and to know the volume of contents removed via the OG tube.
In addition to existing guidelines, ASA should consider adding: Clears x24 hours prior to procedure (then standard NPO guidelines).
Need more education
What will be critical for gastric POC ultrasound is that it is data/outcomes-driven.
As usual, the ASA rushes to make premature guidelines with low/little evidence, which legally binds us, even while new evidence continues to come out.
It is not evidence-based.
Holding one half-life hardly constitutes safety. This is a concern and we have seen patients who have held as recommended with aspiration/full stomach after OGT placement.
It’s the NPO time, more than the hold time, that has a significant impact on the status of an empty stomach.
The ASA guidelines may or may not be respected by other surgical and procedural services and may potentially, by the very wording, hinder and/or alter my ability to provide appropriate care.
Often, patients who follow current guidelines still have a full stomach. Would recommend extending the holding period and always treating these patients with full stomach precautions.
Taken several POCUS courses already.
Need more evidence to change them. The spectrum of variability of symptoms and gastroparesis is too wide right now to be less conservative overall.
If a patient takes a drug 1x/week, waiting a week before an elective case is JUST outside the dosing window. I personally don't think one week is enough time.
Pocus exam is not 100 % for sure at empty.
Per Joshi and Samba, the one-week half-life of Ozempic indicates we should probably hold it for four half-lives.
We need better hard data on outcomes with these drugs in this context - they're so new we don't really understand the perioperative risks.
Must be stronger than the ASA.
It's inadequate - I have seen plenty of EGDs at my hospital where there is visual evidence of undigested food piled up in the stomach.
Holding Ozempic for one week prior to surgery appears not to be based on science. Therefore, I take that as the lowest-quality evidence recommendation that's arbitrary. I perform gastric US in pre-op, which helps me determine an anesthetic approach.
Doubt they will reduce aspiration. It would make more sense to be on a liquid diet for 24 hours.
Concern that the AGA and ASA guidance now appear to differ for GI procedures.
The pharmaceutical industry needs to warn patients of complications and not just make them magic weight loss medications on TV and print media.
Guidance on postponing if in the active onboarding phase, and more guidance on individual symptoms.
I usually place an OG to suction the stomach after intubation if I have a patient with questionable symptoms or who didn't follow the guidelines.
The guidelines are not based on pharmacology but on dosing structure. I think one week for IM is too short. However, realistically, we cannot capture patients earlier than we currently are to [reinforce] instructions to hold, and surgeons' offices are not reliable at my institution for instructing on medication hold practices.
I have no thoughts.
I think gastric ultrasound needs to be taught in residency as frequently as central venous lines. It is becoming that important. I imagine the NPO guidelines in my lifetime will change to become even more patient-specific.
The current guidance is horrible; it doesn't specifically address most of the cases we have to deal with, such as surgery for cancer diagnosis. There needs to be a clearer statement that says patients with cancer or patients who need surgery for a cancer diagnosis should proceed as usual with RSI.
I did not see individual half-lives and common symptoms mentioned in the attached table in the guidance document. Some people, based on that, have treated things like monjauro differently than, let’s say, trulicity. It might be interesting to ask why many are less concerned by the guidance for those other drugs.
Review studies on the accuracy of gastric ultrasound
In these surveys, list the names of the medication, not just the class of medication.
The gastroenterology society posted its own guidelines that differ related to colonoscopy prep cases.
Other societies have slightly different opinions at times, leading to conflict and confusion.
Effectiveness of RSI in mitigating complications
Reconciling with the Society of Gastroenterology recommendations
I [think] one week is probably not enough for those with other risks such as morbid [obesity], uncontrolled diabetes, etc. In fact, [I just] had a patient who had been fasting for over 10 hrs, off GLP for over a week, vomited gastric contents after induction before LMA placement. After intubation, 300 ml in the stomach.
I’m in two ASCs without the availability of U/S
Gastric ultrasound would be highly subjective and open to interpretation, as well as provider-dependent for obtaining images. I imagine there would be difficulty in delineating degrees of "fullness," and then creating a clinical decision-making scenario. What if the ultrasound is non-reassuring, but the patient is symptom-free? And vice versa?
Even if certified, I would not utilize ultrasound. If I were concerned to the point of using ultrasound, I would cancel the case or do an RSI on an emergent case.
More supportive data
Too permissive, 24 hours off is not sufficient. Unconvinced of the value of bedside ultrasound, both in efficiency and in giving a false sense of security. Anyone taking GLP-1 antagonist should be considered full stomach
May help to avoid cancellations
Ultrasound not immediately available
We desperately need more conclusive evidence, followed by stronger language from the ASA to help support this position in our hospital systems.
Regarding gastric ultrasound, I don’t currently use gastric ultrasound to assess aspiration risk in other patient populations with known delayed gastric emptying (DM, anticholinergic meds, etc.) and would need to see data supporting the benefit of assessment over routine RSI for these patients.
Impact of not stopping the meds versus properly stopping them prior to surgery
Your question about which symptoms would have me delay the case is poorly worded. It depends on the circumstances and the surgery involved.
If a patient has been recommended for a GLP1a and is taking it, they generally fall into the highly compliant patient population. I have therefore not seen this to be a consistent problem.
Make them more uniform across all anesthetizing sites
Guidelines somewhat confusing, not very robust in my opinion. Why are the daily ones only held for one day? if weekly ones are taken less than one week, should I cancel or proceed? LMA vs. RSI ETT?
n_total_ = 56

Anesthesiologists’ years of experience showed a weak positive correlation and statistical significance with reading/awareness of the ASA guidance document (correlation = 0.20, Fisher's exact test p = 0.009) and the type of guidance followed (correlation = 0.19, Fisher's exact test p = 0.015). Anesthesiologists’ years of experience showed weak positive correlation and statistical insignificance with responses to: ASA 2023 guidance “preserves patient safety” (correlation = 0.16, Fisher's exact test p = 0.092), frequency of patients receiving proper instruction for holding GLP1-RAs preoperatively (correlation = 0.16, Fisher's exact test p = 0.281), and frequency of patients correctly following instructions for holding GLP1-RAs preoperatively (correlation = 0.13, Fisher's exact test p = 0.822).

The frequency of seeing a patient taking a GLP-1 RA showed strong positive correlation and strong statistical significance with the frequency of patients receiving proper instruction for holding GLP-1 RAs preoperatively (correlation = 0.45, Fisher's exact test p < 0.001) and frequency of patients correctly following instructions for holding GLP-1 RAs preoperatively (correlation = 0.46, Fisher's exact test p < 0.001). The frequency of seeing a patient taking a GLP-1 RA showed a weak positive correlation and statistical significance with reading/awareness of the ASA guidance document (correlation = 0.196, Fisher's Exact Test p = 0.01). Weak positive correlation and insignificance were seen with the type of guidance followed (correlation = 0.14, Fisher's exact test p = 0.32) and responses to the item ASA 2023 guidance “preserves patient safety” (correlation = 0.12, p = 0.64).

## Discussion

Overall, this survey highlighted the ongoing complexity and discourse regarding GLP-1 RAs in the perioperative setting. The majority of anesthesiologists polled in this study agreed that the ASA’s 2023 guidance on GLP-1 RAs in elective procedures preserves patient safety, and most anesthesiologists currently follow this guidance in their practice. However, there were a significant number of anesthesiologists who did not agree that the 2023 ASA guidance preserved patient safety, with free responses detailing the uncertainties posed by the guidance. These uncertainties involved the utility and subjectivity of gastric POCUS, the lack of evidence concerning intraoperative pulmonary aspiration risk in patients taking a GLP-1 RA, confusion regarding GLP-1 RA dosing and half-life, and the difference in guidelines between various medical societies.

Along with the study’s conclusions regarding the ASA’s 2023 guidance, this study demonstrated that most anesthesiologists frequently see patients taking a GLP-1 RA in their practice. Most anesthesiologists experience that patients are properly instructed and correctly holding these medications prior to their procedure. Most anesthesiologists polled in this survey would cancel an elective case if the patient did not properly hold a GLP-1 RA and GI symptoms were present at the time of the procedure.

After the conclusion of this study, the ASA released updated multi-societal guidance in October of 2024 that detailed new recommendations for GLP-1 RAs in elective procedures [19]. This guidance was developed by five societies: the ASA, International Society for the Perioperative Care of Patients with Obesity, American Gastroenterological Association, American Society for Metabolic and Bariatric Surgery, and Society of American Gastrointestinal and Endoscopic Surgeons [20]. A corresponding letter to the editor of Anesthesiology was also published [21].

The new guidance advises anesthesiologists to continue GLP-1 RA medications throughout the perioperative period if patients are at a low risk for delayed gastric emptying and aspiration. The objective is to maintain glycemic and metabolic control for this patient population. If patients are at a high risk for delayed gastric emptying and aspiration, the new guidance advises a 24-hour liquid diet with the possibility of gastric POCUS to estimate aspiration risk. If the risk of aspiration remains high, then the ASA advises referral to the original 2023 guidance for the holding of GLP-1 RA medication and potentially consider the patients as “full stomach” [19].

The 2024 update to the ASA’s 2023 guidance reverses some initial recommendations and maintains elements of uncertainty for anesthesiologists and patients taking GLP-1 RA medications. Future directions of study should investigate the utility of gastric POCUS in the determination of aspiration risk in patients taking GLP-1 RA medication. Additionally, high-quality studies should be performed to identify the rate of aspiration events in patients taking a GLP-1 RA medication compared to patients who do not.

This study has multiple strengths. The fastidious approach to survey item development minimized threats to reliability and validity. The items were very relevant to the topic, and the survey respondents were a sample of the same group of people for whom the study results are most relevant.

There are several limitations to this study. First, a significant number of respondents were represented by the author’s (NCW) practice, biasing the results toward a Midwest private practice setting. Second, we utilized the survey distribution method of snowball sampling to increase the number of responses for our questionnaire. This method of distribution inherently introduces bias into our results. Due to our loss of control over population sampling, it is unknown how many respondents were forwarded the invitation link to respond to the study questionnaire, making it impossible to determine the response rate of our survey. Additionally, snowball sampling produces non-random “clusters” of populations which may not be perfectly representative of the national population of anesthesiologists [22]. Due to our study's use of a forwarding hyperlink, the hyperlink was allowed to be accessed only once per device; however, it was not possible to determine if individuals accessed the survey hyperlink more than once using different devices. It is also not possible to determine if respondents were practicing anesthesiologists, as credentialing was not required to respond. Some respondents may have been physicians in other specialties or non-physician anesthesia providers. 

## Conclusions

This study found that most anesthesiologists surveyed agreed with the ASA’s 2023 guidance document in preserving patient safety for elective procedures. These physicians also report seeing patients on a GLP-1 RA medication frequently in their practice and observe patients correctly following preoperative holding protocols. In the free responses, respondents cited confusion and uncertainty regarding the evidence behind the 2023 guidance, the feasibility of utilizing gastric POCUS, and the need for more conclusive evidence regarding the holding of GLP-1 RA medications. This study revealed the mixed attitudes of anesthesiologists regarding practice recommendations in the 2023 guidance that were subsequently carried into the current 2024 guidance, highlighting the need for more studies aiming at stratifying the intraoperative aspiration risk in patients taking a GLP-1 RA medication and the practice implications for anesthesiologists.

## Appendices

Appendix A

**Table 11:** Checklist for the reporting of survey studies

Section/topic	Item	Item description	Reported
Title and abstract			
Title and abstract	1a	State the word “survey” along with a commonly used term in title or abstract to introduce the study’s design.	x
	1b	Provide an informative summary in the abstract, covering background, objectives, methods, findings/results, interpretation/discussion, and conclusions.	x
Introduction			
Background	2	Provide a background about the rationale of study, what has been previously done, and why this survey is needed.	x
Purpose/aim	3	Identify specific purposes, aims, goals, or objectives of the study.	x
Methods			
Study design	4	Specify the study design in the methods section with a commonly used term (e.g., cross-sectional or longitudinal).	x
Data collection methods	5a	Describe the questionnaire (e.g., number of sections, number of questions, number and names of instruments used).	x
	5b	Describe all questionnaire instruments that were used in the survey to measure particular concepts. Report target population, reported validity and reliability information, scoring/classification procedure, and reference links (if any).	x
	5c	Provide information on pretesting of the questionnaire, if performed (in the article or in an online supplement). Report the method of pretesting, number of times questionnaire was pre-tested, number and demographics of participants used for pretesting, and the level of similarity of demographics between pre-testing participants and sample population.	x
	5d	Questionnaire if possible, should be fully provided (in the article, or as appendices or as an online supplement).	x
Sample characteristics	6a	Describe the study population (i.e., background, locations, eligibility criteria for participant inclusion in survey, exclusion criteria).	x
	6b	Describe the sampling techniques used (e.g., single stage or multistage sampling, simple random sampling, stratified sampling, cluster sampling, convenience sampling). Specify the locations of sample participants whenever clustered sampling was applied.	x
	6c	Provide information on sample size, along with details of sample size calculation.	x
	6d	Describe how representative the sample is of the study population (or target population if possible), particularly for population-based surveys.	x
Survey administration	7a	Provide information on modes of questionnaire administration, including the type and number of contacts, the location where the survey was conducted (e.g., outpatient room or by use of online tools, such as SurveyMonkey).	x
	7b	Provide information of survey’s time frame, such as periods of recruitment, exposure, and follow-up days.	x
	7c	Provide information on the entry process: –>For non-web-based surveys, provide approaches to minimize human error in data entry. –>For web-based surveys, provide approaches to prevent “multiple participation” of participants.	x
Study preparation	8	Describe any preparation process before conducting the survey (e.g., interviewers’ training process, advertising the survey).	x
Ethical considerations	9a	Provide information on ethical approval for the survey if obtained, including informed consent, institutional review board [IRB] approval, Helsinki declaration, and good clinical practice [GCP] declaration (as appropriate).	x
	9b	Provide information about survey anonymity and confidentiality and describe what mechanisms were used to protect unauthorized access.	x
Statistical analysis	10a	Describe statistical methods and analytical approach. Report the statistical software that was used for data analysis.	x
	10b	Report any modification of variables used in the analysis, along with reference (if available).	x
	10c	Report details about how missing data was handled. Include rate of missing items, missing data mechanism (i.e., missing completely at random [MCAR], missing at random [MAR] or missing not at random [MNAR]) and methods used to deal with missing data (e.g., multiple imputation).	x
	10d	State how non-response error was addressed.	x
	10e	For longitudinal surveys, state how loss to follow-up was addressed.	N/A
	10f	Indicate whether any methods such as weighting of items or propensity scores have been used to adjust for non-representativeness of the sample.	x
	10g	Describe any sensitivity analysis conducted.	N/A
Results			
Respondent characteristics	11a	Report numbers of individuals at each stage of the study. Consider using a flow diagram, if possible.	x
	11b	Provide reasons for non-participation at each stage, if possible.	N/A
	11c	Report response rate, present the definition of response rate or the formula used to calculate response rate.	x
	11d	Provide information to define how unique visitors are determined. Report number of unique visitors along with relevant proportions (e.g., view proportion, participation proportion, completion proportion).	x
Descriptive results	12	Provide characteristics of study participants, as well as information on potential confounders and assessed outcomes.	x
Main findings	13a	Give unadjusted estimates and, if applicable, confounder-adjusted estimates along with 95% confidence intervals and p-values.	x
	13b	For multivariable analysis, provide information on the model building process, model fit statistics, and model assumptions (as appropriate).	N/A
	13c	Provide details about any sensitivity analysis performed. If there are considerable amount of missing data, report sensitivity analyses comparing the results of complete cases with that of the imputed dataset (if possible).	N/A
Discussion			
Limitations	14	Discuss the limitations of the study, considering sources of potential biases and imprecisions, such as non-representativeness of sample, study design, important uncontrolled confounders.	x
Interpretations	15	Give a cautious overall interpretation of results, based on potential biases and imprecisions and suggest areas for future research.	x
Generalizability	16	Discuss the external validity of the results.	x
Other sections			
Role of funding source	17	State whether any funding organization has had any roles in the survey’s design, implementation, and analysis.	x
Conflict of interest	18	Declare any potential conflict of interest.	x
Acknowledgements	19	Provide names of organizations/persons that are acknowledged along with their contribution to the research.	x

Appendix B

PubMed Search Strategy

1. “GLP”[Title/Abstract] OR “GLP-1”[Title/Abstract] OR “GLP-1 RA”[Title/Abstract] OR “glucagon-like-peptide”[Title/Abstract] OR “glucagon-like-peptide 1”[Title/Abstract] OR “glucagon-like peptide”[Title/Abstract] OR “glucagon-like peptide 1”[Title/Abstract] OR “glucagon-like peptide-1”[Title/Abstract] OR “glucagon-like-peptide receptor agonist”[Title/Abstract] OR “glucagon-like-peptide 1 receptor agonist”[Title/Abstract] OR “glucagon-like peptide receptor agonist”[Title/Abstract] OR “glucagon-like peptide 1 receptor agonist”[Title/Abstract] OR “glucagon-like peptide-1 receptor agonist”[Title/Abstract])

2. Anes*[Title/Abstract] OR anesthesia[Title/Abstract] OR anaesthesia[Title/Abstract] OR anesthesiology[Title/Abstract] OR anaesthesiology[Title/Abstract] OR “perioperative”[Title/Abstract] OR “peri-operative”[Title/Abstract] OR “preoperative”[Title/Abstract] or “periprocedure”[Title/Abstract])

3. 1 AND 2

4. Limit to human studies
